# Exploring views on long term rehabilitation for people with stroke in a developing country: findings from focus group discussions

**DOI:** 10.1186/1472-6963-14-118

**Published:** 2014-03-10

**Authors:** Nor Azlin Mohd Nordin, Noor Azah Abd Aziz, Aznida Firzah Abdul Aziz, Devinder Kaur Ajit Singh, Nor Aishah Omar Othman, Saperi Sulong, Syed Mohamed Aljunid

**Affiliations:** 1 School of Rehabilitation Sciences, Faculty of Health Sciences, University Kebangsaan Malaysia, Kuala Lumpur, Malaysia; 2 Department of Family Medicine, Faculty of Medicine, Universiti Kebangsaan Malaysia Medical Centre, Kuala Lumpur, Malaysia; 3 United Nations, University-International Institute for Global Health, Kuala Lumpur, Malaysia; 4 Department of Health Information, Universiti Kebangsaan Malaysia Medical Centre, Kuala Lumpur, Malaysia; 5 International Centre of Casemix and Clinical Coding, Universiti Kebangsaaan Malaysia, Kuala Lumpur, Malaysia

**Keywords:** Stroke, Rehabilitation, Focus groups, Family-assisted therapy

## Abstract

**Background:**

The importance of long term rehabilitation for people with stroke is increasingly evident, yet it is not known whether such services can be materialised in countries with limited community resources. In this study, we explored the perception of rehabilitation professionals and people with stroke towards long term stroke rehabilitation services and potential approaches to enable provision of these services. Views from providers and users are important in ensuring whatever strategies developed for long term stroke rehabilitations are feasible and acceptable.

**Methods:**

Focus group discussions were conducted involving 15 rehabilitation professionals and eight long term stroke survivors. All recorded conversations were transcribed verbatim and analysed using the principles of qualitative research.

**Results:**

Both groups agreed that people with stroke may benefit from more rehabilitation compared to the amount of rehabilitation services presently provided. Views regarding the unavailability of long term rehabilitation services due to multi-factorial barriers were recognised. The groups also highlighted the urgent need for the establishment of community-based stroke rehabilitation centres. Family-assisted home therapy was viewed as a potential approach to continued rehabilitation for long term stroke survivors, given careful planning to overcome several family-related issues.

**Conclusions:**

Barriers to the provision of long term stroke rehabilitation services are multi-factorial. Establishment of community-based stroke rehabilitation centres and training family members to conduct home-based therapy are two potential strategies to enable the continuation of rehabilitation for long term stroke survivors.

## Background

Stroke has emerged as the number one cause of long-term disability among adults, that will only continue to affect many societies in the next 20 to 30 years as the world population ages. Advances in medical care have resulted in two out of three stroke patients surviving their acute stroke episode. Due to this trend, the world is witnessing an increase in the prevalence of stroke survivors, thus the number of those living with disabilities [1].

Literature has demonstrated that, as a stroke enters its chronic or long term post-insult phase, there is increased risk of future stroke and functional decline reported at 5.0% and 9.0% respectively [2,3]. These risks occur even among survivors who have achieved full recovery following rehabilitation. Because of these observations, many stroke management guidelines have now advocate for people with stroke to have access to further rehabilitation, as long as they continue to benefit from the services [4-6]. The main purpose of further rehabilitation is to maximise the survivors’ functional independence, facilitate re-integration into the community and enhance participation in life roles [4-6]. Engagement in ‘long term rehabilitation’, refers to rehabilitation as long as stroke survivors need the service and continue to show functional improvement due to the service utilisations, was found to produce favourable effects on physical performance and psychosocial functioning of stroke survivors, whereas its absence was associated with functional deterioration, rehospitalisation and reduction of quality of life of the survivors [7].

Long term rehabilitation interventions for people with stroke can be successfully implemented in developed countries, however the provision of such services is questionable in countries with limited healthcare resources. As these countries are mainly from low and middle income categories, the health care policies are still focusing on acute care services for both communicable and non-communicable diseases rather than restorative services such as stroke rehabilitation services. In several developing countries in which community-based rehabilitation services have been initiated, lack of funding often remains the barrier to maintaining sustainability [8].

Malaysia as a developing country is challenged with similar issues in providing optimum rehabilitation services to people with disability that includes stroke survivors. The delivery and timing of rehabilitation services to stroke survivors are determined by post-stroke duration or pre decided maximum duration for service utilisation, and not based on individual functional needs and recovery as recommended in current stroke rehabilitation evidence based guidelines. Rehabilitation services are normally discontinued at one year post-stroke in most rehabilitation centres, often without transfer of care plan. Factors such as lack of dedicated stroke rehabilitation wards and shortage of trained rehabilitation professionals are also hindering optimal rehabilitation service provision during the acute and recovery stage. As a result, it can be deduced that many stroke survivors may not have reached their maximum recovery despite being in rehabilitation for more than six months. Thus, further rehabilitation may be beneficial in regaining optimal functional recovery. The aims of this study were to explore perceptions of long term rehabilitation among rehabilitation professionals and people with stroke, and identify strategies for the provision of such services.

## Methods

### Study design

This qualitative study utilised focus group discussion (FGD) in an attempt to understand how long term rehabilitation is perceived by rehabilitation professionals and individuals with stroke. Focus groups were used because they provide an opportunity for in-depth discussion between participants with similar and diverging views; therefore, the resulting supportive and argumentative dynamics add to the richness of the dataset [9].

### Study setting

This study was conducted at two university-based health institutions: Universiti Kebangsaan Malaysia Medical Centre (UKMMC) and the United Nations University-International Institute for Global Health both located in the city of Kuala Lumpur, Malaysia.

### Participants

Two focus groups were formed for this study. The first focus group (FG1) consisted of rehabilitation professionals (RP) who were members of stroke rehabilitation teams from two teaching hospitals (UKMMC and University Malaya Medical Centre), one main public hospital (Kuala Lumpur Hospital) and a community stroke rehabilitation centre under the care of the National Stroke Association of Malaysia. Information about the study and invitations to participate were first sent to the managers of the rehabilitation departments of the selected hospitals. The invitations were followed up by telephone, and the managers were asked to nominate a rehabilitation physician, a physiotherapist, an occupational therapist, a speech pathologist and a medical social officer who were currently members of a multidisciplinary stroke rehabilitation team and have had more than five years of experience in stroke care. Assistance from the managers of the rehabilitation department of the hospitals were sought in selecting the professionals as study participants considering that they would better know their staffs’ experience and ability to participate in a discussion. Rehabilitation professionals working on a part-time or a rotation basis, who had less than three years of experience in managing stroke patients were excluded.

Participants for FG2 were selected from a pool of stroke survivors (SS) who had attended rehabilitation intervention in the Rehabilitation Department of UKMMC. None of the researchers were involved in the care of stroke patients at the department, thus the list of the survivors was obtained from a physiotherapist who was in-charged of stroke rehabilitation services at the department. Included participants were those who had had a stroke one or more years prior to enrolment. Stroke severity was also used as a selection criterion in order to gather data from a range of different perspectives; participants were selected from three categories of stroke severity: mild, moderate and severe. Patients’ medical records and physiotherapy notes were reviewed to retrieve information on the patients’ National Institute of Health Stroke Scale (NIHSS) score which was routinely used to classify stroke severity. Eligible stroke patients were contacted via telephone, during which, information about the study was provided and invitation to participate was extended. Stroke patients who were known to have severe depression (assessed with the use of the Hospital Depression Index), poor cognitive function as measured with the Mini Mental State Examination (<24), speech and language difficulties as determined by a speech pathologist who assess the patients with a standardised assessment procedure and unstable medical conditions such as unstable angina, which affect engagement in exercise programmes, were excluded.

The recommended sample size to ensure data saturation and control of the discussion process in a FGD is between 6 and 15 subjects [9]. In this study, 15 participants were selected for the first focus group and 12 participants were recruited for the second focus group. The Research and Ethics Committee of UKMMC approved this study. All participants gave their written and verbal consent.

### Data collection

Data collection took place between February and May 2011. Each group met on two occasions which occurred at one-month intervals. Prior to each session, a topic guide was developed based on literature review and consensus, which included questions relating to satisfaction of current rehabilitation services, beliefs toward long term rehabilitation, and possible approaches for long term rehabilitation services. The groups were moderated by the main researcher (NAMN), who has experience in stroke rehabilitation and was facilitated by a stroke rehabilitation consultant (NAA) and a research assistant (NAOO). The moderator encouraged interactions among the participants and ensured that each group fully discussed each study topic and that each participant had an adequate opportunity to express his or her views. All discussions lasted between 90 and 120 minutes and were audio taped. NAOO took field notes and made participant interaction maps at each of the group meetings. The key issues discussed during the first meeting session were fed back to participants at the beginning of the second meeting to enable respondent validation of emerging themes. The second meeting also served as a platform for further discussions on topics that were not fully addressed during the first meeting, in the researchers’ attempt to ensure data saturation.

All data were analysed using a thematic analysis approach. Audio tapes were first transcribed and the transcripts were reviewed for accuracy. NAMN and NAOO analysed the transcripts independently of each other. Themes were identified by the two researchers by constantly reading and re-reading the transcripts. Interpretations made by the two researchers were then compared and discussed until reaching an agreement. The two researchers also discussed and agreed upon quotations from participants that best represent the themes. The themes that emerged in FG1 were also compared with those arising from FG2 and similar ideas were noted. An independent researcher (DKAS) validated the analysis and the study findings.

## Results

### Demography of participants

All of the fifteen rehabilitation professionals who were selected for FG1 attended the sessions. However, only 8 out of 12 stroke survivors who had agreed to participate attended the FG2 sessions. The reasons for non-attendance of the remaining four survivors included feeling unwell (2 survivors) and having other priorities (2 survivors). The characteristics of all of the participants are shown in Table 1.

**Table 1:** Characteristics of the focus group participants

**Criteria**	**Rehabilitation professionals (N)/years**	**Stroke survivors (N)/years**
Age, years	27-54	30-72
Gender		
Male	5	4
Female	10	4
Occupation		
Rehabilitation physician	3	NR
Medical social officer	2	NR
Occupational therapist	3	NR
Speech pathologist	2	NR
Physiotherapist	5	NR
Experience in stroke rehabilitation, years	3-15	1-2
Stroke severity level		
Mild	NR	4
Moderate	NR	2
Severe	NR	2

*NR,* not relevant.

### The needs for continuity of care

The majority of participants in FG1 and FG2 agreed that stroke rehabilitation services in the country had improved over the current decade. However, they felt that enhancement of the continuity of care for stroke patients, following hospital discharge was needed. Participants also perceived a lack of support system as a main obstacle to continued care.

*“Continuity of therapy outside there* [the hospital] *is very lacking. I agree with that because we have nursing care, we recommend continued nursing, by which I mean home nursing. They will cover a 10 km radius from the hospital, but none of them will go farther away. So, it will be a problem. That’s the major problem for us…that is, the long term care for the stroke patients. It’s the same for follow up patients. If they* [patients] *stay nearby, they can come for follow up, but if they’re far away, we don’t know what happens to them.” (RP1)*

*“Even though we have home care nursing here and before discharge* [from the hospital] *I think we have a stroke conference with the family, but the continuity of care is not there.” (RP4)*

“When we want to develop the rehabilitation programme, we are looking at getting the continuum of care in place you know…and trying to identify what mechanism should be there and in our local setting, do we have enough of a support system? Compared to overseas settings, they are very rich with the support systems, the community centres have their own swimming pools, fitness gyms you know, but it’s not the physiotherapist who conducts the exercise, it’s just a qualified fitness training instructor. In other words, they are so well integrated.” (RP5)

### Beliefs about long term rehabilitation

Most of the participants believed that further rehabilitation for stroke patients was useful provided that the stroke patients are motivated to continue with the therapy. Nonetheless, a few participants from the rehabilitation professionals group were sceptical about the benefits of continued of rehabilitation for chronic stroke patients:

*“For chronic patients, we know they have already reached a plateau, there is no long term potential for these patients. So, it’s* [the focus] *more towards the prevention of complications. So we teach the caregivers of the patients about complications. The issue is that prevention of complications has already been taught by the OT and PT from the beginning. So, the caregivers have already learned all of these.” (RP1)*

*“ I think that…rehab has to stop somewhere. We don’t give rehab until the patients die. Why? After a point, you know…rehab should reach a point. We can only get to that stage* [and not go any further]*, that’s how it is…” (RP2)*

On the contrary, all of the participants in FG2 had positive beliefs about long term rehabilitation. They claimed that they have no problems continuing ‘exercises’ in a longer amount of time post-stroke and viewed long term ‘exercises’ as important to maintaining strength.

“It’s better…..it’s better, the more you exercise, the stronger you will be.” (SS1)

“Okay, no problem. Exercise is definitely good. At the moment I am still doing it.” (SS2)

### Perceived barriers to long term stroke rehabilitation

Participants in both groups raised concerns regarding several limitations that could be seen as barriers to the implementation of continued rehabilitation services for stroke patients. The main barriers were:

#### Uncertainties about the definition and goals of long term stroke rehabilitation

A participant from the rehabilitation professionals group emphasised a need to clearly define ‘long term stroke’ to assist in achieving the targeted outcomes and plan rehabilitation for long term stroke patients.

“Is there an acceptable definition of long term? When? How many years would you consider long term and whether that definition applies in our own country, in local settings?”

“What is the expected rehabilitation outcome for a long term stroke? Definitely there is a difference from rehab outcomes at the different levels. So, that’s why we need to categorise patients. We said long term….. how many years, is it already gone into the chronic phase, and what would then be the expected rehab outcome?” (RP5)

#### Resource limitation

All participants viewed limited resources in the current healthcare system as a major barrier to the provision of long term rehabilitation to people with stroke. Inadequate or ill-equipped stroke rehabilitation wards may be a reason for some patients missing out on rehabilitation after being discharged from acute care in medical or neurology wards. There were also very limited community based rehabilitation (CBR) centres for stroke in this country, which served as a transfer of care destination for patients following hospital-based rehabilitation.

“So, automatically, for patients who ideally meet all of the criteria for rehabilitation, we can try to send them all for rehab. But, some get to miss out because we don’t have a stroke ward.” (RP8)

*“I agree; the continuity of the programme outside there* [the hospital] *is very lacking. So, nobody really… I don’t know who monitors them or if they get improved in that way, maybe there’s continuity. So, hospital-based, healthcare-based, and then what…..?” (RP11)*

The existence of a small number of privately owned CBR centres was of little assistance to stroke survivors due to their high cost, even though the therapy they offer is similar to that provided in public hospitals.

“I went to X College….so expensive….I only went a few times. It’s expensive. Furthermore, what they taught me, I had already learned when I was at the hospital. Nothing new….it’s expensive, I don’t want to continue.” (SS7)

“So expensive…..it’s 200 ringgit a month. I can’t stand it.” (SS6)

In response to a question whether long term rehabilitation could be organised in hospital setting as a solution to the lack of CBR centres, all participants expressed doubts.

“No, there is nothing we can do in the hospital. We can’t keep these patients for the long term just for what is essentially community care. There must be a place where we can actually discharge patients to make sure that they still get the necessary continuous community care. And that is the essential part which is missing.” (RP2)

Participants felt that the current hospital environment would not enable such services to be provided due to several reasons:

#### Shortage of manpower

Staff shortages requiring workers to care for too many patients at once had affected the staffs’ amount of contact time with their patients. They claimed that caring for stroke patients for an extended period for long term rehabilitation would only make this situation worse.

“We don’t have enough staff, we don’t have enough time. And we don’t have enough beds.We can’t keep our patients for so long.” (RP2)

“I was thinking of sending my patients to allied health sciences for further care. But, because the number of staff there is very small, that becomes a problem.” (RP1)

*“The therapists can’t spend enough time* [with each patient] *because there are just too many patients. And because they don’t spend enough time with the patients, patients, when they come, just complain, you know… they spent 40 minutes (for therapy), but they don’t improve. They said ‘the therapists put me on some exercise machine, then they forgot about me. They only come back to me after I am done.” (RP2)*

“But, again here it’s the issue of, you can have the bodies, but if you don’t have the ‘positions’, there will be no bodies to fill them”. (RP5)

Understaffing was also viewed as a main reason for long waiting times, which may have led to poor compliance among stroke patients in attending hospital care and rehabilitation.

*“By the time that we finished seeing them* [the therapists]*, it’s already 1 or 2 pm, then another long line to get the medications. So, it’s a whole day at the hospital if not for lack of parking.” (RP3)*

#### Scarcity of hospital transport services and parking spaces

The issue of poor mobility services was also raised. Living far away from hospital has caused patients with low socio-economic status to not be able to pay for public transport to attend rehabilitation for an extended period of time.

“The patients will be lost to follow up. The fact is that there is a transportation problem or they live too far away.” (RP2)

*“If they* [the patients] *stay nearby they can come follow up, but if they live far away, we eventually lose them and don’t know what happens to them.” (RP11)*

*“It’s not easy for them* [the patients] *to pay to come by cab…so expensive.Now they have to pay about 30 ringgit or more. So, transportation becomes a problem.” (RP2)*

“I stayed in Ampang….In Ampang, to get to a hospital even once, it’s difficult to get a taxi or a bus.” (S2)

Among patients who could afford own transportation, they found the limited parking spaces in hospital area as troublesome*.* In many situations, patients had to park their vehicles outside the hospital’s compound which was of significant distance from the rehabilitation unit.

*“It’s the parking that’s a problem….it’s* [the lot’s] *always full. Sometimes we had to park at the stadium.” (S8)*

“I came alone. Parking was always a problem.” (S1)

“I had to walk all the way. That day, I had to park near the fast food restaurant across the main road and had to walk.” (S4)

Other factors related to the patients and their family members were also identified as barriers to long term rehabilitation for people with stroke:

#### Low awareness among patients and their families regarding optimum rehabilitation

The lack of awareness of the importance of optimum rehabilitation among patients and their families was seen to result in poor compliance to rehabilitation. This was attributed mainly to lack of patient education offered by highly occupied rehabilitation staff.

*“It’s* [the problem] *a lack of explanation from doctors and I think, therapists as well, in a way. We spend less time with them, you know....But, other big problems that I have found is that the lack of us* [rehabilitation professionals] *educating patients of the need for them to come back to physiotherapy and occupational therapy or speech therapy. Some of them get an opinion that, “I just need to come to see the doctor.” That’s what they actually think. But, I think, if we actually sit down with the patients during the whole time and emphasise the need for them to comeback for therapy, then they would come back.” (RP2)*

#### Poor motivation among stroke survivors

The issue of motivation to participate in continuous therapy also emerged in the discussion among the participants in FG2. Two participants who have had severe stroke claimed that their motivation level declined as the stroke became chronic hence were not motivated to continue practicing the previously learnt exercises at home.

“Initially, I was motivated. After several months, I don’t feel that excited anymore.” (S8)

“I like doing exercise at the hospital but at home I feeling lazy. Also because no one is there to guide me.” (S6)

### Approaches to long term rehabilitation

Several suggestions were made by participants in both groups as strategies for long term rehabilitation for stroke patients:

#### Establishing community-based stroke rehabilitation

Participants in both FGs agreed that community-based rehabilitation centres are greatly needed to manage long term stroke patients.

*“What we should have is a rehabilitation centre and a rehabilitation hospital. Patients, after a certain period, they can be transferred to a rehabilitation hospital…. because they really have to* [go there]. *From the rehabilitation hospital, you can either discharge the patient….. or get them to community centres. You should discharge either to a CBR or nursing home.” (RP1)*

“Other than the primary community healthcare, we can also involve community-based rehabilitation settings.” (RP11)

*“I think, the* [CBR] *need to be set up to offer services to patients. We need the centres to manage projects that they can organise.” (RP15)*

*“If we had CBR, we wouldn’t have these* [lack of further care] *problems.” (RP1)*

#### Addressing the issue of manpower shortages

Some of the participants stressed the need to ensure adequate number of physicians and therapists in community-based rehabilitation centres if they were to be established, due to the nature of CBR being multidisciplinary. Creating therapy or rehabilitation assistant positions may be a temporary measure to overcome the issue of the lack of therapists.

“Again here it relates to us having a discussion with the human resources department. Then, I believe they would be willing to work with us…this is again a multidisciplinary team using an interdisciplinary approach.” (RP5)

“This brings up the point of creating rehab assistant positions… whereby these assistants are able to do some of the specific tasks of both roles.” (RP8)

“So, we are offering two types of assistance to patients. One is what we called practical assistance?” (RP14)

#### Optimising family in continuing therapy at home

Another potential approach to increase the continuity of rehabilitation, which was viewed as useful by most participants in FG1, was to involve the family members in conducting basic therapy at home.

“There should be assistance nearby, you know, somebody who can help achieve the patient’s goals.” (RP2)

“…give caregiver training to families of patients who need long term care, especially those who are bedridden. We can give them checklists……the patient’s medications, nursing care, positioning and other things…the therapists can teach all of these tasks.” (RP14)

*“I have seen a few cases in which the family plays their part, and I tried to do the same with our patients during intensive rehab…., you can see progress* [in the patient] *after a few months.” (RP6)*

*“Actually, we can train family members…we can train them. To your question whether we can train them to do this* [assist with basic therapy], *yes…many of them can be taught. And I think if we do this over time…hmm I think we can start doing (this) in acute care…. We should keep teaching this to the family members* [in an on-going basis]*. Every time they come to physiotherapy, the physiotherapists train the caregiver. Essentially they have to go home and do the exercise for one week.” (RP2)*

“Actually for some basics, like for those of us in occupational therapy, basic ADL can actually be taught to family. But, then like I said before, that depends on who the main caregiver is…the one that will actually care for the patient.” (RP7)

*“If you’re talking about* [caregiver training] *yes…yes, the carer can be trained to do this at home.” (RP15)*

Although family-assisted therapy was seen as one possible approach to continuity of rehabilitation, the commitment of family members was questionable. The majority of participants felt that the family of stroke patients had not given adequate support throughout the rehabilitation process, especially in the later stage of stroke recovery.

“What the other thing we can do is train the family members, whether they have enough time or the initiative to do it, that’s another thing to think about. We even can train maids. Maids come and we train maids. But, maids now, they either run away or resign. It’s not easy.” (RP2)

“I agree with Dr S, it’s not easy to train caregivers. Even in the ward we have problems identifying who the main caregiver is. So, it’s difficult for us to train caregivers on what to do at home. Even when we want to do a home visit to see what the patients do at home….to review equipment needed, how they go to the washroom, kitchen, and do other things, the problem is after we have trained the family, they pass the job on to their maid. Again the maid is doing the work.” (RP7)

*“I have seen a few cases in which family members, when the physio comes, they* [family members] *go to one side (corner) of the room, chit chat chit chat..(means talking). So, we cannot see anything, I mean the progress of the patient.” (RP6)*

Children were observed to be too occupied with their own goals, their own work and their own families. Thus, they were not able to care for their sick parents.

*“Time is changing, you know. Children… they need to go to work as well, you know. They take care of their own goals. They have their own families. Children cannot take care of their parents while working; especially now that everything comes at a price. It’s making it* [the situation] *worse. They need more work.” (RP2)*

“In the long term, even if the stroke survivor is suffering from isolation, if they do, you know… there’s no contact, family members just don’t have time.” (RP1)

“The children, sometimes, can’t even do small things. They don’t even know the meaning of stroke. I have a son but, you know, children nowadays, they can’t be bothered.” (SS8)

“ I agree…they don’t know what stroke means. My family was okay initially…but after a while, they will complain. They can nag and say ‘you have been exercising a lot but still not recovered?’ (laughing). That’s what they say.” (SS5)

“The children……through my experience, we can’t rely on them 100%, you know. They may have their own problems, their own stress. To depend on them, I think, that’s not possible, maybe a little. They have their own work, too. So, the only person is the wife.” (SS6)

Participants in FG2 also claimed that family members can be overprotective and that this had discouraged stroke patients from performing home exercises.

“Whenever I do the exercises, my wife will be angry. She will say why did you do it (exercise)? If you fall, who’s going to look after you?” (SS5)

“I wanted to walk outside but my wife scolded me by saying What’s wrong with you? You already had a stroke but still you want to go out.” (SS8)

## Discussion and conclusions

Qualitative research that explores the perspectives of stroke survivors and multidisciplinary healthcare professionals towards long term stroke rehabilitation is scarce. It is not known whether the provision of long term rehabilitation is feasible in the current healthcare environment in most developing countries, despite the increasing importance of the services. Views from both healthcare providers and healthcare users are important prior to planning long term rehabilitation programmes for stroke survivors. We attempted to address these concerns as well as explore issues surrounding the continuity of care for people with long term stroke from the perspectives of rehabilitation professionals and stroke survivors.

Our study revealed gaps in the provision of rehabilitation to individuals with long term stroke living in the community. Participants generally agreed that further rehabilitation beyond that currently organised in the hospital system would benefit stroke patients to achieve optimal recovery. However, materialising such services remains a challenging task. Barriers related to both healthcare providers and patients were identified. Limited healthcare resource was the major issue attributed to the discontinuity of therapy as recognised by the participants in the study.

Understaffing was perceived as a main reason for the lack of opportunities for long term rehabilitation for individuals with stroke in hospital settings. Our study support previous observation of staff shortages as a key barrier to efficient stroke rehabilitation services [10,11]. Woo and colleagues [10], in a study of three rehabilitation hospitals, reported a significant reduction in function among stroke survivors when the number of hospital staffs was reduced over a ten year period. In many developing countries, rehabilitation professional shortage is a real concern and poses a challenge to the healthcare system [12]. In Malaysia, taking physiotherapist as an example; the most recent statistics on human resources in healthcare shows that currently, there are less than 900 registered physiotherapists available in public hospitals in the country [13]. Even if an estimate of 300 physiotherapists in private healthcare institutions is added to this figure, the ratio of physiotherapists to the population is only 1:25,000, which is far below the targeted ratio of 1:15,000 for the country [14]. This is especially striking when compared to ratios of other countries, such as Singapore’s 1:7500 and the UK’s 1:1400 [15]. The lack of staff directly affects the intensity of therapy and the therapist-patient contact time; these are important factors which influence outcome of stroke rehabilitation [11]. In coping with heavy individual caseloads due to staff shortage, many therapists choose to focus mainly on therapy delivery, with little emphasis given to the educational aspect of patient care. Lack of patient and carer education may be the reason for lack of motivation among stroke patients and low commitment among their carer towards on-going rehabilitation, as also raised by participants in this study.

The scarcity of hospital transport and mobility services is a contributing factor to poor access to healthcare among patients who do not own a vehicle, are too ill to use public transportation or cannot afford the cost of the transportation. In hospitals in which mobility services are available, such services are limited to patients who live within a specific distance from the hospital, for example, within a 15 to 20 km radius. Among patients who have a means of transportation, transport costs to access healthcare centres can be substantial. A previous review estimated transportation costs as ranging between 25 and 28% of total healthcare costs, second highest after expenditure on medicine [16]. Our study documented similar issues with regard to transportation, as experienced by the participants. In addition, our participants identified inadequate parking as a hindrance to regular rehabilitation attendance among stroke patients. Our findings are consistent with those of a past study in which, weather, transportation and personal safety were reported as factors that influence physical activity practice among older people [17].

Further therapy for stroke patients who require long term rehabilitation is best organised at a community-based rehabilitation centre [4], so that hospitals can focus on managing the care of acute or sub-acute stroke patients. Meta-analysis has shown that community-based stroke rehabilitation of any kind reduces the incidence of functional decline and maintains or improves activities of daily living in stroke survivors [18]. However, in most developing countries, publically funded community-based stroke rehabilitation facilities are lacking. Malaysia is experiencing a similar situation, leaving stroke patients with no choice other than to seek rehabilitation services that are offered at private health care centres. Nevertheless, some participants in our study claimed that high fees for therapy in these healthcare centres have resulted in discontinuing therapy and potentially low usage of the service among stroke patients in the country.

A potential, low-cost approach for expanding resources available for rehabilitation is to involve family members in conducting specific therapies at home. This approach has been investigated as early as in the 1990’s under a concept of shared responsibility [19] however, in spite of positive outcomes, the approach has not gained popularity. Recently, researchers have again explored the potential of this so-called ‘informal caregivers’ in influencing stroke survivors’ functional recovery. Findings from a qualitative study by Galvin and colleagues [20] strengthened the view that the family has a role to play in the delivery of therapy following a stroke. In their study, family involvement was perceived as enabling carry-over of treatment, improving handling skills and assisting the family unit to cope with post-stroke problems upon discharge. These ideas were tested in a randomised controlled trial of a family-mediated exercise programme involving forty stroke patients, and it was found that family members can successfully implement therapy when training is provided by a qualified therapist [21]. Our findings support these studies; they indicate the potential of family-assisted therapy at home as a solution to enable continued care for long term stroke patients, especially following discharge from hospital rehabilitation.

While family involvement can be optimised to facilitate on-going therapy for stroke survivors at home, a concern of the level of commitment among family member was raised. There is a need to explore family members’ readiness to exercise their role as a ‘therapy facilitator’ and the extent to which they are able to participate prior to implementing any kind of family-assisted rehabilitation interventions. A previous study found that, despite expressing willingness to be involved in the delivery of unsupervised therapy, family viewed work commitments, lack of confidence and unsuitable treatment times as factors that would limit their participation [20]. Careful therapy planning, in terms of intensity and time and adequate training for the family to increase their confidence level as care-giver is thus crucial in planning a family-assisted therapy.

Our study has several limitations. Although recruitment of participants was carefully attempted based on the individual’s ability to express their ideas, some participants did not engage adequately in the discussions. Several participants in both FGDs had to be encouraged to express their opinion. This has interrupted the natural flow of the discussions and threatened the richness of the data. This would also imply that the two focus groups with a total number of four discussion sessions may not be adequate in ensuring saturation of data. A larger number of focus groups and recruitment of new participants for both the rehabilitation professional and the stroke survivors groups would be required to sufficiently explore this topic. There were also issues related to low voice volume in some participants, resulting in difficulty in transcribing and interpreting the audio-taped data. Field notes taken during each discussion session has somewhat been useful to compliment data obtained from the recorded conversation.

In spite of these limitations, the findings of our study have implications for the provision of rehabilitation services to long term stroke patients. Our study demonstrated that there is poor continuity of care for stroke patients as the patients enter the chronic phase of recovery as well as following discharge from hospital-based rehabilitation. The lack of continued care is multi-factorial; this includes a shortage of rehabilitation personnel, poor awareness among patients and caregivers due to insufficient patient education, unsupportive caregivers and a lack of community-based rehabilitation facilities for stroke patients. Planning rehabilitation for long term stroke patients must address these factors to ensure effective service delivery. Considering the increasing number of stroke incidence and, thus, people with disabilities in the future, it is timely that community-based rehabilitation services are set up to enable continued care and further rehabilitation for stroke patients upon discharge from hospital-based care. By having more CBR centres, rehabilitation services can be delivered as close as possible to people’s homes and communities thus reducing drop-outs from therapy due to mobility and transport problems. In the absence of this centre, given adequate training, the role of family can be optimised in a home-based therapy as an alternative approach to further rehabilitation.

Despite these recommendations, question remains on what would be an ideal timing of ‘long term rehabilitation’ for stroke survivors. It is generally understood that longer duration of rehabilitation would mean longer consumption of health care facilities thus higher health care cost. Recent studies have shown that due to brain plasticity, functional recovery is possible at many years following a stroke although neurology recovery is observed to reach maximum level within three months post-insult [22]. Evidence-based stroke rehabilitation guidelines recommend that stroke survivors should have access to rehabilitation as long as they benefit from the service [4-6]. However, no suggestion is provided about the overall duration of rehabilitation; rehabilitation professionals are left to use their own judgement in determining when to discharge stroke patients under their care. Future research that look into this concern is warranted to guide decision making related to long term rehabilitation service to people who unfortunately had a stroke.

## Competing interests

The authors declare that they have no competing interests.

## Authors’ contributions

NAMN designed and conducted the study, performed the analysis and drafted the manuscript. NAA advised on the study design, facilitated data collection and revised the manuscript. SS advised on the study design and coordination. AFAA helped coordinate the study and assisted in data collection. NAOO assisted in data collection and data analysis. DKAS validated the analysis findings and revised the manuscript. SMA advised on the overall conduct of the study. All authors read and approved the final manuscript.

## Pre-publication history

The pre-publication history for this paper can be accessed here:

http://www.biomedcentral.com/1472-6963/14/118/prepub
